# Trommer’s Extract of Malt

**Published:** 1880-09

**Authors:** 


TROMMER'S EXTRACT OF MALT.—Our acquaintance with this valuable preparation began several years ago, and we were among the first of the professsion to prescribe it frequently in Baltimore.

Time has tended only to strengthen the favorable opinion we expressed in a letter to the manufacturers of Trommer's Extract of Malt, a few years ago. Of the conditions of the system in which it may be used with profit, it is unnecessary for us to speak editorially, as the professsion is aware of the great value of malt in the successful treatment of debility, and many forms of actual disease.

